# Genome sequence of equine influenza virus isolated from horses in southeast Kazakhstan in 2020

**DOI:** 10.1128/MRA.00433-23

**Published:** 2023-10-12

**Authors:** Yelizaveta Khan, Yermukhammet Kassymbekov, Symbat Suleimenova, Kobey Karamendin, Temirlan Sabyrzhan, Sardоr Nuralibekov, Klara Daulbayeva, Abdikalyk Abishov, Moldir Akhmetzhanova, Nurlan Akhmetsadykov, Zhanat Batanova, Aidyn Kydyrmanov

**Affiliations:** 1 Research and Production Center for Microbiology and Virology, Almaty, Kazakhstan; 2 Virology laboratory, Almaty, Kazakhstan; 3 Kazakh National Agrarian Research University, Almaty, Kazakhstan; Queens College Department of Biology, Queens, New York, USA

**Keywords:** EIV, gene, horse, H3N8, influenza virus A, sequence, virus

## Abstract

An influenza virus strain, A/equine/Almaty/268/2020, was isolated from horses in southeast Kazakhstan in 2020. Here, we present the nearly complete genome sequence of this epidemic strain. This study was aimed at obtaining the complete genome sequence of the isolate.

## ANNOUNCEMENT

Equine influenza (EIV) is an acute respiratory infection caused by type A virus that belongs to the family Orthomyxoviridae and genus *Alphainfluenzavirus* that spreads rapidly, despite the availability of commercial vaccines (1). There are two main subtypes: H7N7 and H3N8, which were isolated from horses (2). The A/H7N7 virus is thought to have disappeared since 1979 (3). The viruses currently circulating in horses worldwide belong to the H3N8 subtype (4).

The EIV strain A/equine/Almaty/268/2020 was isolated from the PCR-positive nasal swab of sick horse during the outbreak of respiratory infection among them in Almaty suburb village (Kazakhstan) in September 2020. Viral RNA was extracted from nasal discharges with the QIAamp Viral RNA Mini Kit (Qiagen). Reverse transcription-polymerase chain reaction was performed with the set of oligonucleotide primers for simultaneous differential diagnosis of the influenza A virus from equine herpesvirus rhinopneumonia (5). The isolation of EIV from PCR-positive samples was carried out by inoculation of the sample into the allantoic cavity of chicken embryos (6). The genomic libraries for sequencing were prepared using the NEBNext Ultra RNA Library Prep Kit for Illumina. The RNA fragmentation was carried out to sizes of about 400–450 bp by an enzymatic method using bivalent cations as part of the kit. Sequencing was performed using the MiSeq Reagent V.3 kit v.3 on MiSeq (Illumina).

In total, 1,879,282 raw sequencing reads per sample were obtained, with a mean length of 268 nucleotides per read. The obtained sequence data were trimmed at the 3′ and 5′ ends with an error probability limit of 0.05, assembled, and mapped with the installed Geneious Prime (Biomatters, New Zealand) mapper (medium sensitivity, four iterations) against the eight segments of the genetically close strain A/donkey/Shandong/1/2017 (H3N8) (GenBank No. MG132044 to MG132051) using the default parameters of Geneious Prime software. In the final assembly, the obtained genome of A/equine/Almaty/268/2020 was 13,365 nucleotides in length.

The gene coverage of the studied virus ranged from 1 to 1,411 nucleotides, while the average coverage was 801 nucleotides. BLAST analysis of eight genes showed high homology (97.55%–99.28%) with modern Asian EIV. HA gene was 98.71% similar to the strain A/equine/China/Ulumuqi/2015, MK215815.1 (Table 1).

**TABLE 1:** Comparison of the nucleotide sequences of all genes of the Kazakhstan influenza A strain with the genetically most closely related strains in GenBank

Gene or segment	Size (nucleotides)	GC content (%)	Most closely related strain	Identity with most closely related strain at nucleotide level (%)	GenBank accession no.
PB2	2,283	42.4%	A/equine/China/Ulumuqi/2015	97.55%	MK215812.1
PB1	2,274	41.9%	A/equine/China/Ulumuqi/2015	98.86%	MK215813.1
PA	2,340	40.7%	A/equus caballus/Arkhangai/2/2007	98.59%	MH796252.1
HA	1,704	39.3%	A/equine/China/Ulumuqi/2015	98.71%	MK215815.1
NP	1,499	45.5%	A/donkey/Shandong/1/2017	99.06%	MG132048.1
NA	1,413	40.3%	A/equine/China/Ulumuqi/2015	98.51%	MK215817.1
M	988	47.2%	A/equus caballus/Ulaanbaatar/4/2011	99.19%	MH796328.1
NS	840	40.7%	A/donkey/Shandong/1/2017	99.28%	MG132051.1

Comparison of BLAST analysis of gene segments A/equine/Almaty/268/2020 showed reassortment of EIV circulating in Central Asia in 2007–2020.

**Fig 1:**
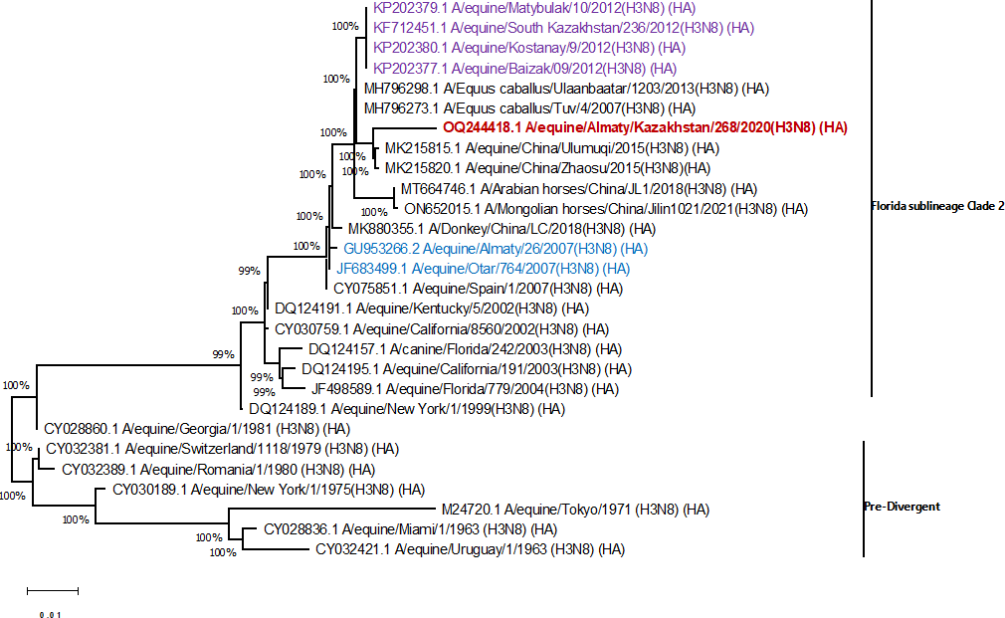
Phylogenetic tree, full-length coding sequence of the HA gene A/equine/Almaty/268/2020 in the public domain, representatives of two genera of H3N8 equine viruses. The trees were created using the maximum likelihood method (MEGA 11 software) and confirmed by the neighbor-joining (NJ) method (using the Tamura-Nei model with 500 bootstrap replicates). Kazakhstan strains, highlighted in colored fonts.

For the phylogeny characterization of the hemagglutinin gene, the phylogenetic tree of HA gene was constructed using the neighbor-joining method in MEGA 11.0 (7) (Fig. 1). This analysis involved 28 nucleotide sequences. There were a total of 1,633 positions in the final data set.

Phylogenetic analysis showed that the A/equine/Almaty/268/2020 H3N8 isolate belongs to the Florida sublineage 2 clade and is very similar to that previously circulating in China.

## Data Availability

The complete genome sequence of A/equine/Almaty/268/2020 is available at GenBank under the accession numbers OQ244415 to OQ244422. Raw sequence reads were deposited under BioProject accession number PRJNA955388 and BioSample accession number SAMN35100499.
